# Design and Implementation of a Low-Cost Chlorophyll Content Meter

**DOI:** 10.3390/s23052699

**Published:** 2023-03-01

**Authors:** Zacharias Kamarianakis, Spyros Panagiotakis

**Affiliations:** 1 Department of Electrical & Computer Engineering, Hellenic Mediterranean University, 71410 Heraklion, Greece; 2 Institute of Agri-Food and Life Sciences, University Research Center, Hellenic Mediterranean University, 71410 Heraklion, Greece

**Keywords:** chlorophyll meter, SPAD, atLeaf, Arduino, chlorophyll content, low-cost, chlorophyll sensor, sensor clip, 3D printing, portable device

## Abstract

Chlorophyll meters are portable devices used to assess and improve plants’ nitrogen management and to help farmers in the determination of the health condition of plants through leaf greenness measurements. These optical electronic instruments can provide an assessment of chlorophyll content by measuring the light passing through a leaf or by measuring the light radiation reflected from its surface. However, independently of the main principle of operation and use (e.g., absorbance vs. reflectance measurements), commercial chlorophyll meters usually cost hundreds or even thousands of euros, making them inaccessible to growers and ordinary citizens who are interested in self-cultivation, farmers, crop researchers, and communities lacking resources in general. A low-cost chlorophyll meter based on light-to-voltage measurements of the remaining light after two LED light emissions through a leaf is designed, constructed, evaluated, and compared against two well-known commercial chlorophyll meters, the SPAD-502 and the atLeaf CHL Plus. Initial tests of the proposed device on lemon tree leaves and on young Brussels sprouts plant leaves revealed promising results compared to the commercial instruments. The coefficient of determination, $R^{2}$, was estimated to be 0.9767 for the SPAD-502 and 0.9898 for the atLeaf-meter in lemon tree leaves samples compared to the proposed device, while for the Brussels sprouts plant, $R^{2}$ was estimated to be 0.9506 and 0.9624, respectively. Further tests conducted as a preliminary evaluation of the proposed device are also presented.

## 1. Introduction

It is well known that leaf chlorophyll concentration is most accurately determined and measured by using analytical chemistry methods that incorporate the extraction of chlorophyll in a solvent and the performance of the subsequent measurements using a laboratory spectrophotometer [1,2,3]. Widely used solvents for chlorophyll extraction include acetone, ethanol, methanol, diethyl-ether, dimethyl-formamide (DMF), dimethyl-sulphoxide (DMSO), and chloroform, with the first three solvents mentioned being the preferred choice among the others [4]. Extinction coefficients of the preferred extraction solvent are then used in conjunction with the spectrophotometric equation for the conversion of absorption values to chlorophyll concentration [1,2,3,4]. Estimating the amount of chlorophyll in plant leaves is a fundamental prerequisite for many non-destructive techniques, efficient nitrogen fertilizer management, and plant growth monitoring studies. Such techniques usually incorporate the use of leaves’ digital photography and image analysis [5,6], flatbed color scanners [7], aerial photography, remote sensing and hyperspectral imaging [8,9,10,11,12], artificial neural networks combined with color models [13], smartphone applications combined with neural networks and image processing [14,15], leaf color charts [16,17], combined different imaging modules and high-throughput phenotyping image processing techniques, such as the fusion of visible (RGB), hyperspectral, and fluorescence imaging of plant leaf data [18], optical methods combined with low-cost vision-based approaches [19], miniaturized systems for the simultaneous measurement of leaf total chlorophyll content and chlorophyll a/chlorophyll b ratio [20], to name a few. However, non-destructive optical techniques have also systematically been used in an effort to achieve a rapid, relative indication and determination of chlorophyll concentration, due to their practical characteristics that are advantageous for the management of crops, such as the direct availability of results in the field, fast acquisition times, and potential for periodically monitoring the crop [21,22]. For this purpose, handheld optical chlorophyll meters have been widely used, with the SPAD-502 (Konica Minolta, Inc., Tokyo, Japan) [23] and its predecessor model SPAD-501 from the same manufacturer, the CCM-200 (Opti-Sciences, Inc., Hudson, NH, USA) [24], and the atLeaf CHL Plus [25] (FT Green LLC, Wilmington, DE, USA) being the most well studied. Other instruments that have been introduced for similar studies, with some of them providing an assertion of equivalent functionality at a considerably reduced or similar expense, include, among others, the N-Tester that is almost undifferentiated to SPAD-502 (Yara International ASA, Oslo, Norway) [21,26], the Dualex 4 Scientific (Force-A, Orsay, France) [27], the CL-01 Chlorophyll meter (Hansatech Instruments Ltd., Pentney, King’s Lynn, Norfolk, United Kingdom) [28], the MultispecQ V1.0 (PhotosynQ Inc., East Lansing, MI, USA) [29,30], and the MC-100, which features a similar hardware design and principles of operation as the Opti-Sciences CCM-200 (Apogee Instruments Inc., Logan, UT, USA) [31]. In Table 1, an indicative, but not exhaustive list tabulates some of the transmittance-based, hand-held optical chlorophyll meters and sensors that can be found in the market nowadays.

Most of the aforementioned instruments measure the transmission of radiation through a plant leaf at two different wavelengths; more specifically, wavelength peaks approximately centered at the range 640–660 nm and 930–940 nm for the red and near-infrared (NIR) electromagnetic spectrum measurements, respectively. Substantially, chlorophyll has “standard absorbance” peaks in the blue (400 to 500 nm) and red (600 to 700 nm) regions, with no or negligible absorbance in a portion of the NIR band (e.g., at 800 to 1000 nm). To exploit this feature of chlorophyll, usually most of the chlorophyll sensors measure the absorbances of a plant leaf specifically in the red and NIR regions. The measured transmittance through the leaf at the red wavelength, in the proximity of 650±10 nm, closely approximates the region where the extinction coefficient of chlorophyll a equals that of chlorophyll b, so most chlorophyll meters, for example the SPAD-502 or the atLeaf CHL Plus, calculate estimated values of the relative total chlorophyll content from leaf transmittance [32,33,34,35] in this wavelength region (e.g., peak center at 650 nm in the case of the SPAD-502 [23] and 640 nm [25] in the case of atLeaf CHL plus). Increased chlorophyll content in the measured sample leaf area leads naturally to a significant absorption of red radiation (e.g., this is the reason that to the human eyes most plants appear green), while the NIR radiation measurement is typically used as a reference wavelength, since the plants transmit most of this kind of radiation. As an output, the hand-held chlorophyll meters calculate index values that specify the relative chlorophyll content in a leaf at two different wavelengths, the index band, which is a region where chlorophyll absorption takes place (e.g., RED-band), and the infrared band (IR-band), which is considered as a reference band used to account for differences in path length and for compensation purposes [32,36,37,38,39,40,41,42]. In the IR band, negligible or very low amounts of the incident IR radiation is absorbed, a fact that is mainly due to leaf water content [34,35]. These measured values in leaves are dimensionless values and are usually expressed in SPAD units, in the case of Minolta SPAD-502; atLeaf units, for the case of atLeaf CHL Plus; or in the percentage transmittance ratio, which is the Chlorophyll Content Index (CCI) value, specifically for the case of CCM-200. Both the output values in SPAD units and atLeaf units, as well as the CCI values, are based on calculations using either a logarithmic ratio, for the first two instruments, or a simple ratio for the latter one (Equations (1) and (2)) for the light transmission through a leaf at two wavelengths. These equations are as follows:

(1) $$CCI=\frac{\%\;transmission\;IR_{\left(931\right)}\;}{\%\;transmission\;RED_{\left(653\right)}}=\frac{I_{IR\left(931\right)}^{\prime }/I_{IR\left(931\right)}}{I_{RED\left(653\right)}^{\prime }/I_{RED\left(653\right)}\;},$$

(2) $$SPAD\;\left(or\right)\;atLeaf=k\times log\left(\frac{\%\;transmission\;IR_{\left(940\right)}}{\%\;transmission\;RED_{\left(650\right)\;or\;\left(640\right)}}\right)+C\approx k\times log\left(CCI\right)+C,$$

where $I_{IR}^{\prime }$ and $I_{RED}^{\prime }$ are the measured leaf light transmission intensities at the specific (in each measurement) infrared and red wavelengths and $I_{IR}$ and $I_{RED}$ are the light intensities of the IR LED light source and the RED LED light source (both different among the devices), respectively. The numbers in the parentheses in the equations above indicate the nm where the transmission of radiation from the respective LED is centered. For example, SPAD-502 measures radiation centered at 940 nm and 650 nm, while the atLeaf CHL Plus measurements concern LED light radiation centered at 940 nm and 640 nm, respectively. Similarly, the output CCI values of the CCM-200 are the ratio of transmission of radiation from an IR LED peak centered at 931 nm to the transmission of radiation from a RED LED peak centered at 653 nm. The left side of Equation (2) above is expressed in its more general form as it was provided by [40]. The approximate equality on the right side of Equation (2) holds, since both the SPAD values and CCI (but also the atLeaf values) are all based on a ratio of the transmission at closely related wavelengths [36,39]. However, similar expressions have been reported in the literature that mainly consider the slope or gain calibration coefficient *k* as unity, or similarly the intercept (offset) coefficient *C* as zero. In any case, these calibration coefficients (the gain *k* and the offset *C*) are different among the chlorophyll meters (e.g., the SPAD-502 and the atLeaf CHLE Plus) as they are different in their output values. Moreover, these coefficients have not been released by the manufacturers [32,33,36,40]. 

The primary objective of this study was to develop, from scratch, a portable, low-cost, yet accurate device, capable of estimating non-destructively the relative chlorophyll content of plant leaves, while achieving the performance of recognized commercial portable instruments, such as the SPAD-502 and the atLeaf CHL Plus, that we focused on in this work. Towards this direction and based on the similar design principles of these two well-known commercial chlorophyll meters, we evaluate the possibility of replicating their behavior by means of their output with our low-cost developed one, we compare their system functionality, their reliability and measuring reproducibility, and investigate the correlation between them. Our correlation analysis with these devices utilizes Equation (2) above in this work. However, the methods presented can be applicable for similar investigations with other instruments if available, too. The main contributions of the present work are twofold: the first is the detailed presentation of the device’s design and construction where, together with the initial experiments presented, we believe that they will be useful for similar, low-cost, experimental, yet worthy implementations; the second is the experimental and customizable design approach followed, which instead of the final outcome being a computing, relative chlorophyll estimation “black-box”, it permits the display and recording of all basic parameters involved (e.g., of each LED transmission through a sample and not just the final measurement). Although it appears a trivial task, these intermediate measured values can be valuable, since they can be used and compared with other chlorophyll estimation research efforts that use simulated or field-acquired data on the relevant wavelengths. Moreover, since our device is a two-part design, with minor modifications the measurement clip can be removed and replaced with a similar mechanism targeted to different or more demanding measurement applications than the ones presented in this work (e.g., measuring the chlorophyll content of oregano’s herb leaves or even thyme’s).

Initial tests of the proposed device on lemon tree leaves samples and on young Brussels sprouts plant leaves revealed promising results compared to the commercial instruments. The experimental results show the estimated coefficient of determination, $R^{2}$, to be 0.9767 for the SPAD-502 (*RMSE* = 0.055) and 0.9898 for the atLeaf CHL Plus (*RMSE* = 0.0366) in the case of lemon tree leaves samples compared to the proposed device, while for the Brussels sprouts plant, $R^{2}$ was estimated to be 0.9506 and 0.9624 for the SPAD meter (*RMSE* = 0.0243) and atLeaf meter (*RMSE* = 0.0212), respectively. Further tests conducted for preliminary evaluation of the measurement accuracy and repeatability of the proposed device, but also of the other two sensors used in this study, are also presented. Moreover, these latter experimental results, on measurements performed using low-cost plastic color sheets and gel color correction light filters, show the estimated coefficient of determination to be $R^{2}=0.9940$ (RMSE=0.0232) for the case of atLeaf and similarly a $R^{2}=0.9746$ (RMSE=0.0476) for the SPAD-502, as compared to our sensor. The measuring accuracy and repeatability of the proposed device, expressed in terms of atLeaf and SPAD units, were calculated to be ±1.34 and 0.2999 atLeaf units for the case of atLeaf CHL Plus, while for SPAD-502 they were calculated to be ±1.22 and 0.2780 SPAD units, respectively. 

The rest of the manuscript is organized as follows: Section 2 presents the proposed device’s design and construction in detail; the experimental results together with discussion and comparison with previous works are detailed in Section 3; finally, Section 4 provides a future outlook and concludes the manuscript.

## 2. Materials and Methods

### 2.1. Experimental Design

**Figure 1:**
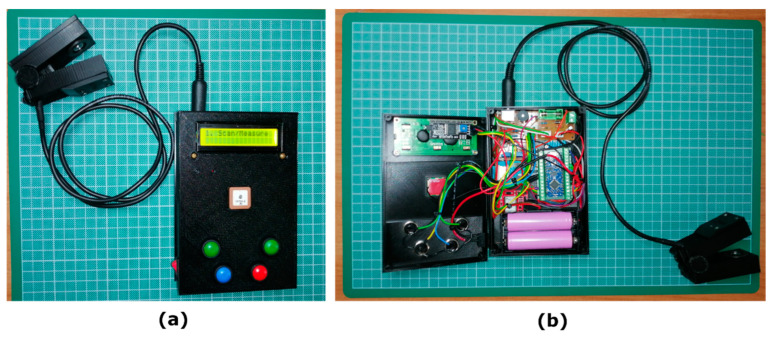
The low-cost chlorophyll meter presented in this work: external (**a**) and internal view (**b**).

In the present work, a low-cost chlorophyll meter (Figure 1a,b) was constructed using 3D-printed hardware and off-the-shelf electronic materials. The whole system design comprises two main housing parts: (a) the sensor clip part that is responsible for the leaf samples’ placement and measurements and (b) the “control box” that is a constructed electronic unit that houses the microcontroller together with the various necessary microelectronics that take part in data acquisition, data processing, data logging, storage, etc. All the housing components were constructed using an open-source fused deposition modelling 3D printer (Original Prusa Mini+, Prusa Research a.s., Prague, Czech Republic), although another 3D printer could have served the purpose as well.

**Figure 2:**
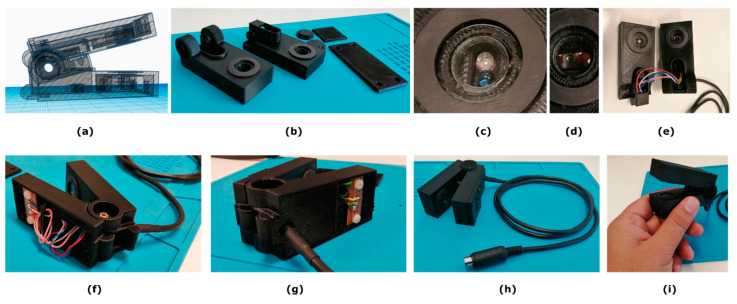
The experimental prototype sensor clip part of the device. Figures (**a**–**i**) show details from the concept design stage to the final implementation.

The sensor clip part (Figure 2) is a movable structure that houses in its upper part (dimensions WxLxH: 33 mm × 76.3 mm × 23 mm) the two selected transmittance 3 mm LEDs; a Vishay TLDR4900 red LED of dominant/peak wavelength 648/650 nm, respectively, and a Kingbright L-34F3BT NIR LED of peak wavelength 940 nm (Figure 2c). The lower part (dimensions WxLxH: 33 mm × 78.9 mm × 27 mm) houses a low-noise, high-sensitivity light-to-voltage converter (TAOS TSL257-LF [43]) which was placed directly at the opposite side of the LEDs for the attenuation measurements of each LED emission through a leaf sample. This optical voltage converter combines a photodiode and a transimpedance amplifier on a single monolithic CMOS integrated circuit. Its output voltage is directly proportional to the light intensity on the photodiode. Both the upper and the lower 10 mm circular openings were firmly sealed with one completely transparent, plastic PET laser-cut circular disc, 1 mm in thickness, attached to each hole (Figure 2c–e). One rubber black flat O-ring, 1.9 mm height, was hooked up to each side (Figure 2b) in order to facilitate the leaf placement but also in order to create a small chamber isolated from ambient light during the measurements. Both O-rings are of the same internal ($D_{in}=15\;\mathrm{mm})$ and external ($D_{in}=23\;\mathrm{mm}$) diameters. Finally, a small compression–return spring of 5 mm outer diameter and 0.5 mm stainless steel wire was placed between the dedicated slots, designed on both parts (Figure 2e) to keep the sensor clip open. 

When the sensor clip is fully closed, with the leaf inserted in its tip, all measurements are performed in full darkness (e.g., only the device’s LED lights are involved in the measurement procedure). With the arrangement just described, leaves of maximum thickness ~1.5 mm can be measured. Although thicker samples can easily fit between the sensor-clip’s upper and lower parts, these parts remain completely parallel when fully closed, up to this maximum leaf thickness just mentioned. The construction details of the developed experimental sensor clip part of the device from the concept design (Figure 2a) to its final implementation (Figure 2h,i) are shown in Figure’s 2 panel. The total weight of the plug-and-play constructed sensor clip, along with its 1 m long cable with the MiniDIN 6-pin connector, is 102 g. The optical voltage converter TSL257-LF, placed in the lower part of the clip (Figure 2d), delivers broad spectral sensitivity across and beyond the visible spectrum and more specifically in the region between 300 nm and 1100 nm. This fact, in conjunction with each selected LED that had a peak emission wavelength falling within the spectral sensitivity of the detector, ensures that the optical measurements achieve the maximum efficiency, since there is a good match between the detector response and the emission spectra of each light source. Moreover, it is important to note, and in accordance with the manufacture’s technical notes [43] related to the irradiance responsivity parameter, that among the tested emission wavelengths, at 645 nm this photodetector produces a higher sensitivity compared with other wavelengths (i.e., 428 nm, 470 nm, and 565 nm). Hence, it was important to set the output voltage of the detector to a standard initial voltage prior to measurements, so that the attenuation degree for both LED illuminations through the leaf could be impartially compared. For this purpose, the system calibration was performed with the sensor clip fully closed and by adjusting the intensity of both LEDs until the output voltage of the detector was set to a lower threshold (e.g., 4.6 V). This procedure was performed to ensure that the maximum possible detected intensity through the medium, that is the air in this case, was lower than the detector saturation level, that is 5 V.

**Figure 3:**
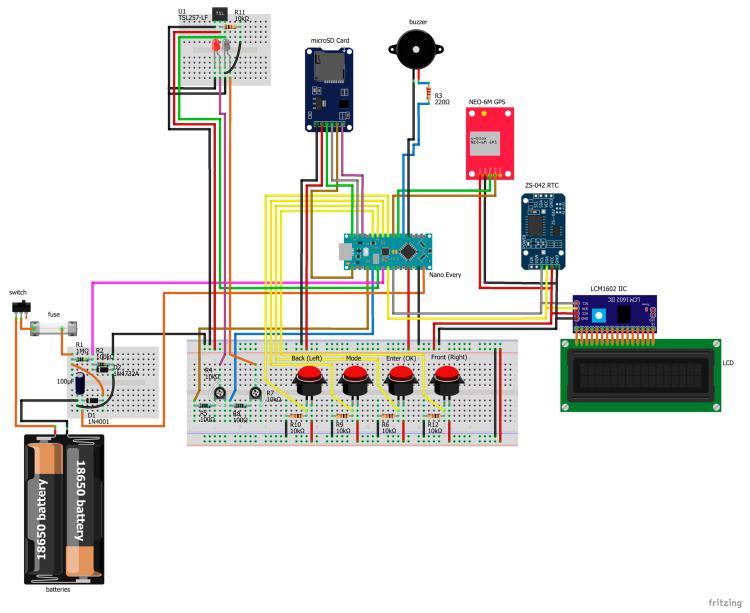
The wiring diagram of the experimental chlorophyll meter device’s prototype.

The circuit design was mainly built around an 8-bit, 20 MHz microprocessor (Model ATMega4809; Microchip Technology Inc., Chandler, AZ, USA) that was housed on an Arduino Nano (Arduino Nano Every board, Arduino CC), although an equivalent board could have also served the purpose. The microprocessor was programmed in the C-based Arduino language. The output data values are displayed on an inexpensive 16X2 LCD module with a Hitachi-HD44780-compatible controller that is driven with a low-cost, address-changeable I2C serial interface board module. Other modules included in the design are a Micro SD storage board with SPI interface driver together with an I2C bus interface, Real Time Clock (RTC) module (DS3231 AT24C32) for read/write operations needed for data logging on a MicroSD card storage device, and a UART GPS receiver module (NEO-6MV2) with a u-blox 6 positioning engine and an integrated antenna. The power supply circuit includes a few typical electronic components that are a voltage divider comprising two 1% accuracy resistors in front of a typical 1N4001 Schottky power diode, as well as a 5.6 V 1N4734 Zener diode acting as a voltage clamp in front of the divider, and a decoupling electrolytic 100 μF capacitor that suppresses potential high transient effects. The portable low-cost device prototype is powered by two rechargeable lithium-ion 18,650 batteries, rated at 3.7 V each (4.2 V at full charge). Battery drains tests indicated that the device, with fully charged batteries, can withstand daily 1.5 h sampling for approximately three months. The batteries’ power monitoring is performed through voltage average measurements on the voltage divider’s output side circuit that feeds an analog input pin of the microcontroller. This way, voltages greater than the normal range between 0 and 5 V can be measured (e.g., the 8.4 V DC voltage of both batteries when fully charged) continuously and with adequate precision in real time. The set of voltage divider resistors, together with the output 5 V ADC reference voltage from the Arduino microcontroller’s regulator, were calibrated using a digital multimeter and a stable power supply. The detailed electronics components’ wiring diagram is shown in Figure 3. 

**Figure 4:**
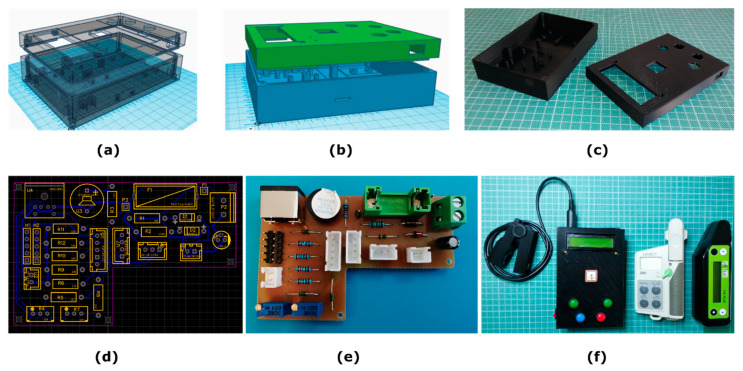
The designed model’s front (dimensions-WxLxH: 100 mm × 149.18 mm × 17 mm) and back cover (dimensions-WxLxH: 100 mm × 149.18 mm × 29.02 mm) of the “control box” are shown in (**a**,**b**), along with the 3D-printed parts (**c**). The designed (**d**) and constructed (**e**) simple, one-sided, auxiliary PCB is shown. In (**f**), the experimental meter is shown along with the two commercial chlorophyll meters, the SPAD-502 and atLeaf CHL Plus.

On the front cover (Figure 4a–c), the device has an on/off power switch, the GPS antenna, and four momentary push buttons (Figure 4f) that facilitate navigation through user menus to initiate the sample scanning, data navigation, bad measurements rejection, measurements averaging, basic GPS coordinate’s view, and battery health monitoring. The whole device, just to provide a clue for the construction materials’ expense, can be built using 3D-printed parts and electronic materials at a total cost of roughly EUR 50 (excluding the cost of the rechargeable batteries and that of the micro-SD card).

Finally, for the purpose of connecting all the required modules and the electronic components of the experimental chlorophyll meter device, a simplified circuit and a one-sided Printed Circuit Board (PCB) were designed using the EasyEDA software (Figure 4d). In the prototype experimental design, the Arduino Nano Every board was mounted on a screw terminal breakout kit. Later versions of the design (not presented in this work) include the integration of the microcontroller as well as the use of SMD electronics in the same PCB design, in order to save space in the final implementation and to make the device more lightweight and more compact. Nevertheless, for interfacing all the devices with the PCB, male/female JST XH 2.5 mm connectors were used, because they proved to be ideal for the task by providing a plug-and-play interface and a high-density mounting of the various electronics devices used (Figure 4e). The prototype chlorophyll meter model has a total weight of 405 g, without the batteries included. In Figure 4f, the experimental meter is shown along with the two commercial chlorophyll meters referred to in this study.

### 2.2. Device’s Operation and Software Pipeline

Upon the system’s power up (by turning the ON/OFF switch, located on the left side of the electronic control unit) it subsequently takes about 5 s for the initialization of all the parameters and verification checks of the internals (connected devices verification test, sufficient batteries, etc.). Prior to leaf measurements, the user ought to ensure a zero measurement (sensor clip empty and fully closed) and externally adjust slightly the calibration linear multiturn potentiometers, located underneath the top cover, if needed. The calibration can be achieved using a precision screwdriver. The front cover of the device features two 2 mm holes on the left side and below the LCD for that purpose (Figure 4b,f).

**Figure 5:**
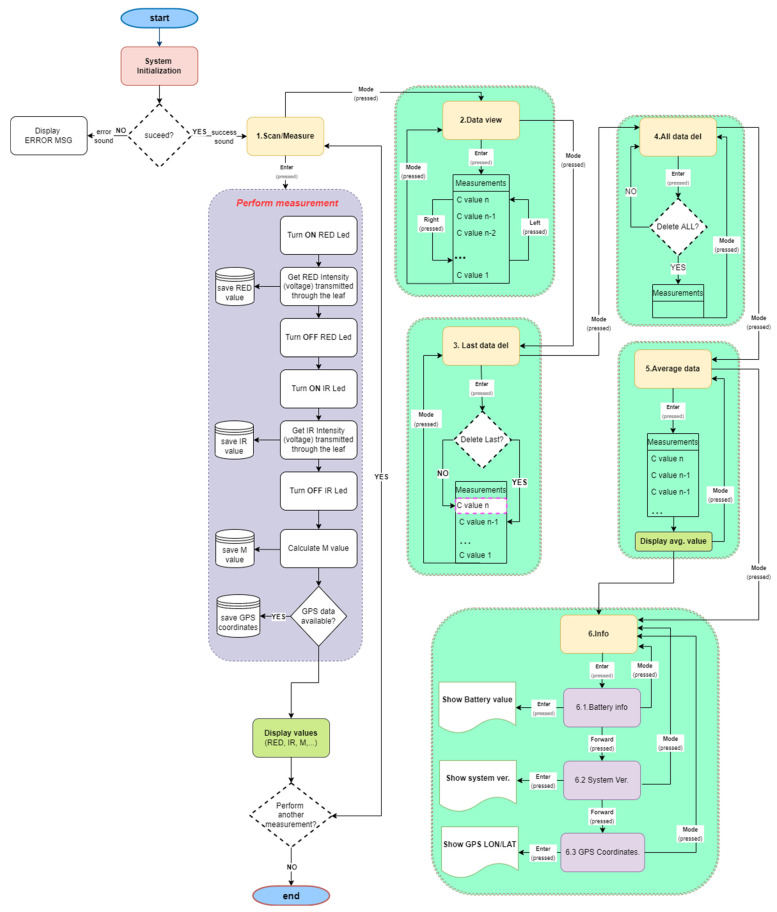
Flowchart of the proposed chlorophyll meter device software’s operation pipeline.

These “zero-measurements” are necessary for air transmission recording of both LEDs. The “air transmission” values are recorded in persistent memory (EEPROM). In a future version, the calibration procedure could be performed automatically by the replacement of the analog potentiometers with digital ones, so manual adjustments might not be necessary. Nevertheless, by pressing the “scan” button in order to perform a leaf measurement, the two LEDs are sequentially turned on and off. After each LED transmission, the light-to-voltage optical converter sensor outputs a voltage that is directly proportional to the light intensity, detected on the photodiode. As a basic filtering scheme, the microprocessor sums 10 analog sample values from the sensor every 10 μs interval, and stores each independent average voltage value, which represents the transmission through the sample or the air, at the specific moment. The two intermediate transmission values of red and infrared LED, along with the calculated decimal logarithmic ratio of both percentage transmittance measurements (e.g., the final output value of the device), are displayed in a fast sequence on the LCD, with the final output value remaining on the LCD. All recorded values are rounded to two decimal places. This way, the user is also being visually informed during measurements of the independent LED transmissions and is provided calibration feedback, or operations such the most recent data rejection, if needed. In addition, the microprocessor converts the final decimal logarithmic output value of the device to SPAD and atLeaf units using, if available, empirically determined calibration routines (details below) stored in persistent memory, and also displays the results on the LCD. Furthermore, all these five values are stored in separate fields in the SD card and, along with the two “empty sample/air measurements”, the day/month/year/time data logging info, the number of current leaf measurement, as well as the GPS coordinates and a checksum value, constitute the current measurement’s stored records (Figure 5). 

The three buttons, named “mode”, “left”, and “right”, facilitate a user’s navigation through the menus that include data measurement view, last data, all data measurement deletion, data averaging, GPS coordinates, and battery life info display, etc. Audible sounds provided during the device’s operation facilitate the overall system’s use (e.g., error alerts, confirmation of successful measurement, deletion command executed, etc.) The flowchart describing the main operation of the device is shown in Figure 5.

### 2.3. Data Acquisition

#### 2.3.1. Data Acquisition on Leaves

**Figure 6:**
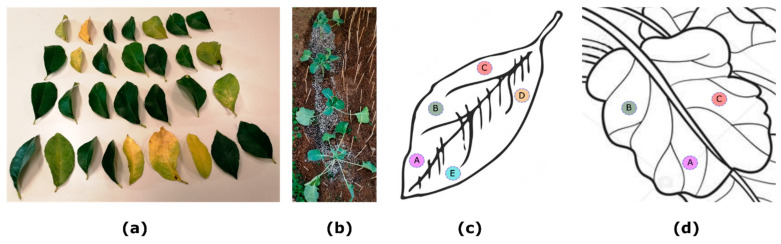
In (**a**), the 30 lemon leaves samples of experiment 1 are shown, selected to span a wide range of color shades (slightly yellow to dark green). In (**b**), a snapshot of some of the young Brussels sprouts growing in an experimental garden that were used for the measurements referred to in experiment 2. The chlorophyll meter measurements were all performed on-site, using all sensors and trying to avoid the major veins and to reach the leaf areas marked with the letters A to E in Figure (**c**) and A to C in Figure (**d**), in most cases. In each of these areas, 5 measurements were acquired, and the values were averaged prior to model fitting. The samples in (**a**) were collected to be photographed after being measured on-site.

The relative chlorophyll meter measurements using the 3 sensors were conducted once for each of the species referred to in this study: on 5 September 2022, for the measurements on 30 lemon tree (*Citrus limon*) leaves (Figure 6a), and on 1 November on 32 young Brussels sprouts (*Brassica oleracea,* Gemmifera cultivar group of cabbages) plant leaves (Figure 6b). The samples were measured on-site, on a small experimental garden of Hellenic Mediterranean University’s facilities (Latitude: 35.31842, Longitude: 25.10303). Averaged readings from the low-cost chlorophyll meter for each sample in the sets were regressed on both averaged readings from the commercial chlorophyll meter devices to develop the calibration curves presented below. Since both commercial instruments exhibit similarities in their working principle, a primary objective of these experiments was to replicate the behavior of each commercial instrument’s output with our low-cost developed one to perform a comparative analysis among the different chlorophyll sensors in order to compare their systems’ functionality while investigating the correlation between them. Having the calibration curves available, the individual readings of transmittance measurements retrieved with the chlorophyll meter proposed in this work can be stored in persistent memory, and further used if needed for converting units between the instruments and the LCD display.

Since we are not aiming for plants’ growth or N status monitoring in relation to leaf chlorophyll content in this study, the chlorophyll meter measurements were just commenced and finalized without repetition, in the specific dates previously mentioned and between 10:00 to 14:00 solar time, for each experiment. All the leaves samples were measured on-site. All the sensors were zeroed prior to each measurement date. The proper functioning of SPAD-502 was verified by using the standard calibration plate and following the instructions of the manufacturer. Additionally, its compensation value was verified to be zero. Similarly, the proposed device was checked frequently for proper functioning and was zeroed accordingly, by using the calibration potentiometers. This procedure, if needed, is performed with the sensor clip empty and fully closed, pressing the “Scan” button and adjusting the intensity of both LEDs until the output detected voltage of each (displayed individually on the LCD) is set to a lower threshold (e.g., 4.6 V). We observed that this procedure needed to be performed twice during the whole samples’ measurements and only for adjusting the intensity of the IR LED. This can be attributed to the ambient temperature changes that may have affected the potentiometer’s initial fine-tuned value. However, this is noted and is reserved for future investigations and updates. Nevertheless, regarding the third instrument, the atLeaf meter is factory-calibrated and according to the manufacturer does not need any special zeroing. The measurements were obtained by clipping the sensor onto the leaf each time. The approximately same position, on the same leaf, was measured 5 times in the leaf areas marked with the letters A to E, in Figure 6c for the case of lemon leaves. For the case of Brussels sprouts, 5 measurements were performed in 3 different areas (Figure 6d) since the leaves were significantly smaller than those of the lemon tree. The measurements with the 3 sensors were performed in the following order: atLeaf CHL Plus, SPAD-502, and then the proposed device.

The five data values that were acquired in the same spots were averaged prior to the statistical analysis for each of the meters. Although it is not a trivial procedure to locate the exact same spot on a leaf during measurements and alternate the sensors during the experiments, we found it to be easier in some cases to use a leaf puncher on-site (only for the case of Brussels sprouts leaves) to extract small 9.5 mm in diameter circular leaf disks from the dedicated areas to be measured. In these cases, the samples were measured immediately after the extraction. Independently from that, during all the experiments, efforts were made to properly align the measurement samples inside the clip of each of the three sensors with the detector axis. For this purpose, the reference notches provided in both the commercial chlorophyll meters proved to be convenient. The measurement area for the three sensors compared is a rectangular window of 2 mm × 3 mm sides for the SPAD-502, and a circular one of 13 mm^2^ for the atLeaf CHL Plus. Similarly, the measurement area of the proposed device is a circular window of 78.5 mm^2^. The integrated photodiode active area of the light-to-voltage converter is round in shape and 1.766 mm^2^ [43].

#### 2.3.2. Data Acquisition on Non-Leaves Samples

**Figure 7:**
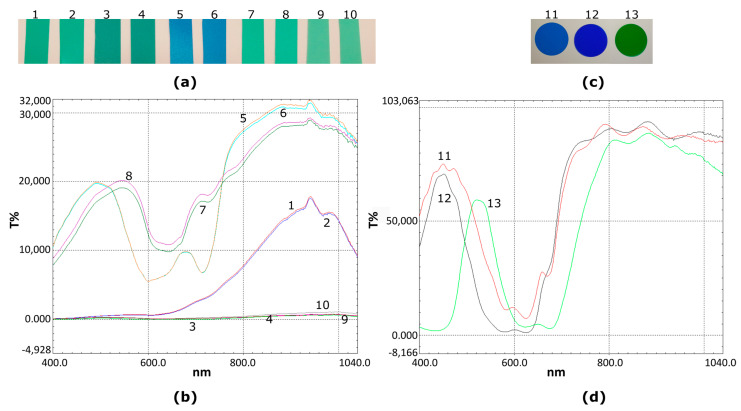
Color plastic filters in 5 different shades of green (no. 1, 2, 3, 4, 7, 8, 9, 10 in (**a**) and 13 in (**c**)) and 3 different shades of blue (no. 5, 6 in (**a**), 11, 12 in (**c**)) used for a low-cost evaluation of accuracy and repeatability of the 3 sensors. In (**b**,**d**) the transmission spectra of the samples are shown as acquired with a spectrophotometer. The numbers in the transmission spectra correspond to the numbers of the sample color filter used.

To evaluate the measurement accuracy and repeatability of the 3 sensors used in this study quickly and with a low cost, a number of measurements were performed on non-leaves samples of different thicknesses but that are quite uniform in transmittance values among their surfaces so that the outcomes would be as independent as possible from the most representative measure or at the exact same measurement position. The main idea was to be able to evaluate the accuracy and repeatability of the 3 devices’ output values in way that is independent of a leaf’s complicated structure and its further “alive” and sophisticated internal mechanisms that potentially can influence the measurements acquired, even “in the exact same spots”. This latter task is moreover extremely difficult to achieve while alternating the devices during measurements. For this experiment, we used 10 low-cost rectangular plastic color sheets and similarly 3 gel round color correction light filters, acquired from the local market (shown in Figure 7a,c). The transmittance spectra in the range of 400–1040 nm of each color filter were measured with a sampling interval of 2 nm in the laboratory using a Shimadzu UV-2401PC, UV-VIS, spectrometer (Figure 7b,d).

The measurement spectra of interest include the range 640 nm to 660 nm for the red radiation and, similarly, the 940 nm for the infrared, that the 3 m utilize. The color plastic filters used, although not constructed from a specialized hard-to-find or costly material, seem to permit a small amount of transmittance at these wavelengths. In Figure 7 above, a visual representation of the transmission spectra acquired with the spectrophotometer is presented, also numbered in accordance with the relevant green or blue color filter used. The samples numbered 1 to 10 are visually similar between successive pairs (e.g., no. 1 and 2 are the same, so are 5 and 6, and so on). However, there are some slight differences in the transmittance spectra as revealed in Figure 7b. The thickness of each pair of the rectangular filters from left to right in Figure 7a is 0.19 mm, 0.31 mm, 0.44 mm, 0.46 mm, and 0.33 mm, respectively. The thickness of each circular disk filter on the right (Figure 7c) is 0.07 mm. By using each of the 3 sensors being compared in this study, 5 measurements were performed in 10 randomly selected positions in each sample, providing a 650-values dataset for each case. The 5 measurements in the same positions were averaged prior to regression.

### 2.4. Device Accuracy Evaluation and Validation Metrics

As trivial statistical analysis indicators of the proposed device’s performance, the coefficient of determination (*R^2^*) and the root-mean-squared error (RMSE) were mostly used in this work. The *R^2^* metric provides one measure of “goodness” of fit by indicating a measure of the degree of fitting between the independent and dependent variables (in this case, the commercial devices versus the proposed experimental one, respectively). The RMSE metric is used to account for the differences between the estimated and observed values. The *R^2^* metric falls between 0 and 1. The higher the value of *R^2^*, the better the model is at predicting the data (e.g., the more variability is explained by the linear regression model). Conversely, the lower the RMSE, the closer the estimated value is to the measured one and the lower the total estimated error. 

Prior to fitting a specific model to our acquired data using the 3 sensors and examining the possibility of the existence of a linear dependence in the datasets, we initially investigated if a linear relationship could be established between our device and each of the commercial instruments. For this purpose, the Pearson correlation coefficient matrix (r) was formed, considering a pairwise variable combination of N average readings of 5 measurements per point, acquired with each sensor (Equations (3) and (4)). This is a 2-by-2 matrix.

(3) $$r=\left(\begin{array}{cc} 1 & \rho \left(A,Β\right)\\ \rho \left(B,A\right) & 1 \end{array}\right),$$

(4) $$\rho \left(A,Β\right)=\frac{cov\left(A,B\right)}{\sigma_{A}\sigma_{Β}}=\frac{1}{N-1}\overset{N}{\underset{i=1}{\sum }}\left(\;\frac{\bar{A_{i}-\mu_{A}}}{\sigma_{A}}\;\right)\left(\frac{{Β}_{i}-\mu_{B}}{\sigma_{Β}}\right),$$

In the equations above, the *A* variable refers to the measurements with SPAD-502 (and similarly with the atLeaf instrument), the *B* variable indicates the measurements with our sensor, ρ(A,Β) is the correlation coefficient of variables A and B, $\mu_{A}$ and $\mu_{B}$ are the mean of A and B, and $\sigma_{A}$, $\sigma_{Β}$ are the standard deviations, respectively. N is the number of measurements with each sensor and cov(A,B) is the covariance matrix of *A* and *B.* The correlation metric ρ(A,Β) is another way, except for the scatter plots, to quantify the strength of a linear relationship between the two variables *A*, *B* datasets.

Finally, we employ the mean and standard deviation metrics to be used for the estimation of the accuracy and the repeatability of all three sensors used in this study applied to non-leaves samples measurements (Section 3.1). More precisely, the accuracy range of each sensor is calculated as the global minimum and maximum values of the difference of each dataset from the global mean (average) value in this dataset. The mean value in the dataset (selection of 10 different random areas, with 5 repetitive measurements in these areas) is considered as the ground truth for this dataset. We examined 9 datasets, with 450 measurement points in total with each sensor because a subset of the 13 color filters was finally used. The repeatability is calculated through the standard deviation that provides the dispersion of measurements in the same 450 measurement points dataset relative to its mean.

## 3. Results and Discussion

Programming for data analysis, regression, and visualization of the results was performed using Matlab^TM^, ver. 2018b software (The Mathworks, Inc., Natick, MA, USA).

The calculated correlation coefficients matrices for the case of SPAD-502 and atLeaf as compared to our device and for all the four samples’ datasets examined are presented in Table 2. In this table, $r_{1}$ represents the correlation coefficient matrix formed considering the measurements acquired with the atLeaf instrument and our sensor, while $r_{2}$ refers to the measurements with the SPAD-502 and our sensor, accordingly. In Table 2 above, two cases for the plastic color filters were discriminated: one considering only the 9 green-colored filters and the other considering all the 13 filters dataset. Nevertheless, it is evident from the results that since the values of the correlation coefficients on both matrices and for all cases examined are too close to 1, there is a strong indication that the measurements acquired with the different instruments in pairs can be linear-correlated. We note that there is a slight precedence of atLeaf CHL Plus instrument correlation with our device, especially in examining the case of lemon tree leaves as well as that of green filters, but also the correlation with SPAD-502 for the same datasets. In any case, the results in all cases are a positive indication for the linear model fitting that was applied and is presented thoroughly in the following Section 3.1 and Section 3.2.

### 3.1. Experimental Resuslts on Non-Leaves Samples—Accuracy Repeatability Evaluation

**Figure 8:**
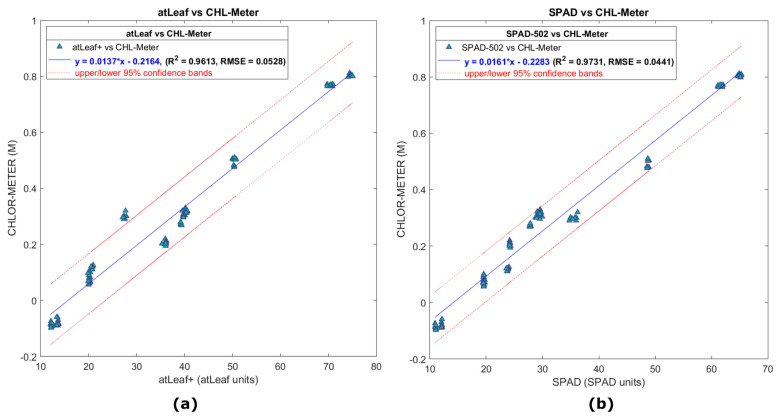
In (**a**), a scatter plot showing the relation of data measurements with atLeaf CHL Plus and (**b**) with SPAD-502 as compared to the proposed sensor, on all 13 green and blue color filters.

Figure 8a,b show plots showing the relationship of the data measurements with atLeaf CHL Plus, SPAD-502, and the proposed sensor in the case of all 13 green and blue color filters. Of the total 650 measurements acquired with each instrument in each filter (10 random areas, 5 measurements per area which were averaged), the plots show the regression of the resulting 130 averaged values datasets used in each case. The values readings for atLeaf ranged from 12.1 to 75.1 atLeaf units, the values of SPAD-502 ranged from 10.9 to 65.4 SPAD units, and the proposed device output range was −0.09 to 0.82, also depicted in the plots.

Although the correlation coefficient in both cases is quite strong ($R^{2}\geq 0.96)$ in both linear regressions, it can be observed that the measured values with atLEaf in the case of the dark blue filter, no. 12, fall out of the 95% confidence bands. Since we are aiming in this section to evaluate by low-cost means the accuracy and repeatability of the three sensors, we decided to further discard all the blue filters’ values and proceed with the green one subset (no. 1, 2, 3, 4, 7, 8, 9, 10, and 13) instead. 

**Figure 9:**
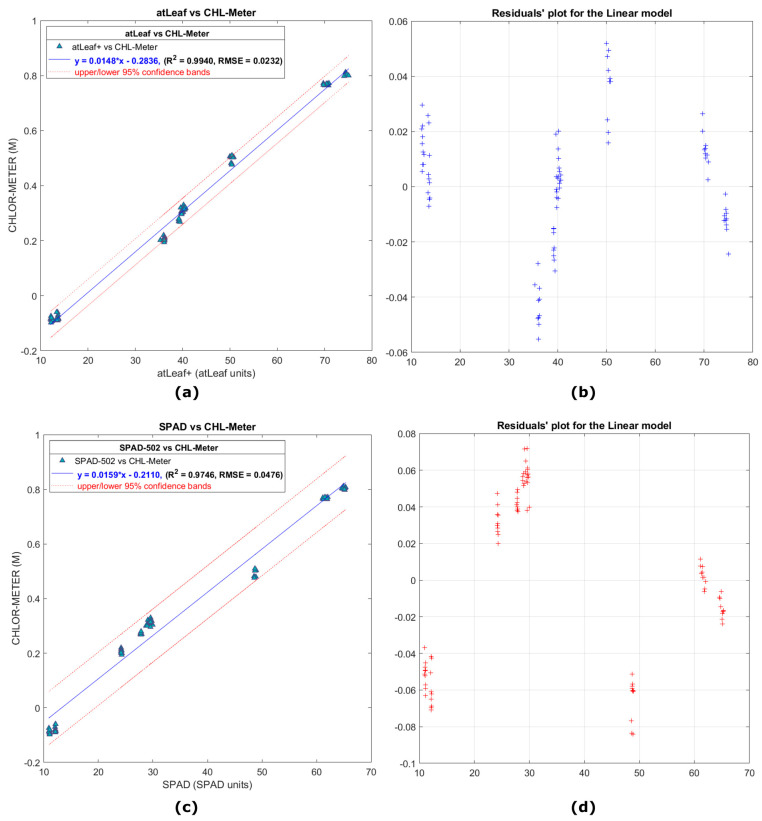
In (**a**), a scatter plot showing the correlation of data measurements with atLeaf CHL Plus and in (**c**), with SPAD-502 as compared to the proposed sensor, with only the 9 green color filters (numbered as 1, 2, 3, 4, 7, 8, 9, 10, and 13 in Figure 7) used. (**b**,**d**) Plots of the residuals (marked with + in the Figure) for the simple linear regression model fit applied on data and plotted on (**a**,**b**). The norm of the residuals was calculated to be 0.2205 and 0.4520, for the atLeaf and SPAD-502, respectively.

In Figure 9a,c the scatter plots show the relation of 90 averaged points measurements (450 readings, of 9 filters on 10 random locations) with atLeaf CHL Plus and SPAD-502 as compared to the proposed sensor, for the case of the 9 green filters. The relevant residuals plots are depicted on the right panel of Figure 9, on Figure 9b and Figure 9d, respectively. Most of the measured values this time fall well within the 95% interval bounds. This is a positive indication and a strong possibility (95% chance) that a new similar measurement will fall within the bands. Unfortunately, at the time of performing this study, we did not have available more green filters for further tests. We note this for in-progress investigations, so the dataset possibly will become denser and also will span a wider range of values. Nevertheless, for the purposes of this study we consider the data provided from the nine green filters to be adequate. 

The two linear regression equations (presented also in Figures above) describe a fit to the data having a strong correlation coefficient $R^{2}=0.9940$ and RMSE=0.0232 for the case of atLeaf and similarly a $R^{2}=0.9746$ and RMSE=0.0476 for SPAD-502. This was expected from the initial correlation coefficient matrices analysis (presented in Table 2) prior to fitting the specific model. It is not uncommon to use filters for meters comparison, as was done in this study. In [44], the authors suggested using Wratten filters for meter comparison. In [35], the measured transmittances of Roscolux plastic sheets were used in the estimation of ***k*** calibration coefficient value of SPAD-502. Similarly, in this study we found the use of plastic filters to be a convenient, fast, and low-cost way to achieve measurement accuracy and repeatability of the sensors. As pointed out in [40], several acquisition parameters can affect the transmission of data through a leaf such as the leaf side (adaxial or abaxial), the non-uniform chlorophyll pigments content, the light-dependent chloroplast movements, scattering and sieve effect [32], the proportion of leaf veins and the flatness of the leaf [33], leaf water content [21,45], but also environmental conditions [45,46,47], such as the rain and dust, just to name a few. So, for the purpose of a quick evaluation of the measurement accuracy and repeatability of the sensors while the outcomes are not being influenced in a great percentage by some of the aforementioned factors, we proceeded with the green filter analysis. Moreover, due to their relatively good color uniformity, the measured values would be as independent as possible from the most representative measurement or at the exact same measurement position.

**Figure 10:**
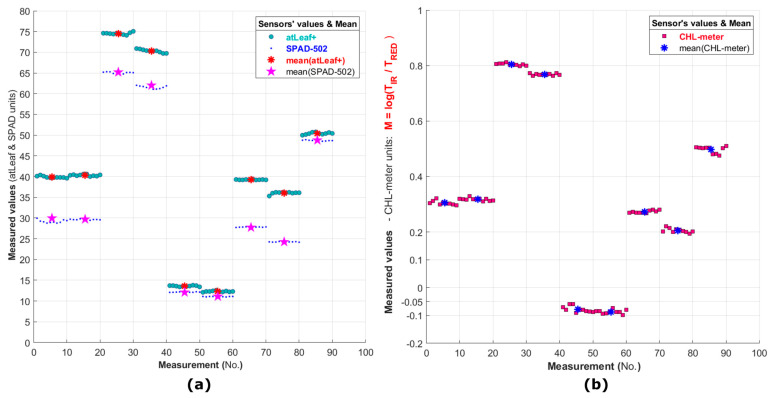
In Figures (**a**,**b**) the repetitive measurements on the 9 green-only color filters (numbered no. 1, 2, 3, 4, 7, 8, 9, 10, and 13 in Figure 7) are shown. In (**a**), the measurements with atLeaf and SPAD are plotted while in (**b**) the same measurements with the proposed device are shown. The numbers on the plot correspond to the filters presented in Section 2.3.2, Figure 7. The red and blue asterisks as well as the magenta star represent the mean of the 10 values plotted for each filter. Each value plotted is the mean of 5 measurements per point.

In Figure 10a,b, a visual representation of the 90-averaged values dataset of the 9 green filters (total 450 total measurements with each sensor) are depicted together with the respective mean value for each filter (the colored asterisks and star) measurement dataset. The plotted 90 points represent an average of 5 repetitive measurements performed on the same point in each color filter for the 10 areas randomly selected. The difference of the 5 averaged values in each of these 10 areas from the mean is considered as an estimation of the accuracy of the sensor in this work.

**Figure 11:**
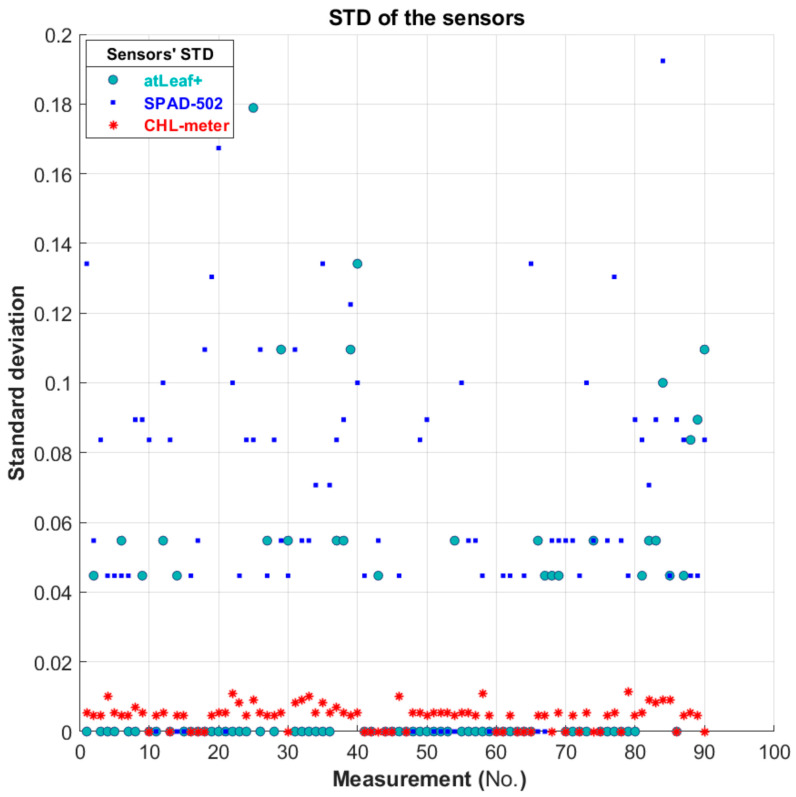
Plot showing the standard deviation among each of the measurements performed, with the 3 sensors used, on the 9 green filters only (90 points in total, 10 random areas per filter, 5 measurements per point).

In Figure 11, a visual representation of the standard deviation among the repetitive measurements performed with the three sensors is depicted. An estimation of the overall repeatability of each sensor was made possible through further analysis of the whole dataset.

In Table 3 below, the evaluation results of the accuracy and the repeatability of both commercial sensors, compared to our proposed device, are presented. Except from the calculated estimated values concerning the evaluation of both accuracy and repeatability in this study, the manufacturers’ relevant specifications are also tabulated for an immediate comparison. Conversion equations (concerning the non-leaves samples regressions and listed in Table 4 in the next Section 3.2) were utilized for converting units between the devices so that the estimation of our device’s outputs (the dimensionless CHL-meter units) could also be expressed in atLeaf and SPAD units. The estimated accuracy of the proposed device expressed in atLeaf and SPAD units is ±1.34 and ±1.22, respectively. Similarly, its repeatability is estimated to be 0.2999 atLeaf units and 0.2780 SPAD units. In Table 3, these quantities are also represented in our device’s relevant metric units.

It can be observed that our estimations regarding the accuracy and the repeatability of both commercial instruments fell within the specifications range provided by their manufacturer, although our experimental dataset used for the evaluation in this experiment spanned a slightly wider and shifted range than the one provided in the manuals. For example, the performance metrics reported in [23] for calculations using SPAD values ranged between 0.0 and 50.0 for the SPAD-502 m, while we used experimental measured values from 10.9 to 65.4 SPAD units. Similarly, atLeaf’s instrument repeatability is reported only for green, valued 50.2±0.1 atLeaf units [25], while in our evaluation we used experimental data in the range of 12.1 to 75.1 atLeaf units. Nevertheless, experimental data subsets as reported in the manuals were tested and the results (not shown in the table) were equivalent to those presented. We conclude that our proposed experimental device demonstrates satisfactory accuracy and repeatability as expressed in its own measurement system but also as compared with the two well-known commercial instruments that we used.

### 3.2. Experimental Results on Leaves Samples

**Figure 12:**
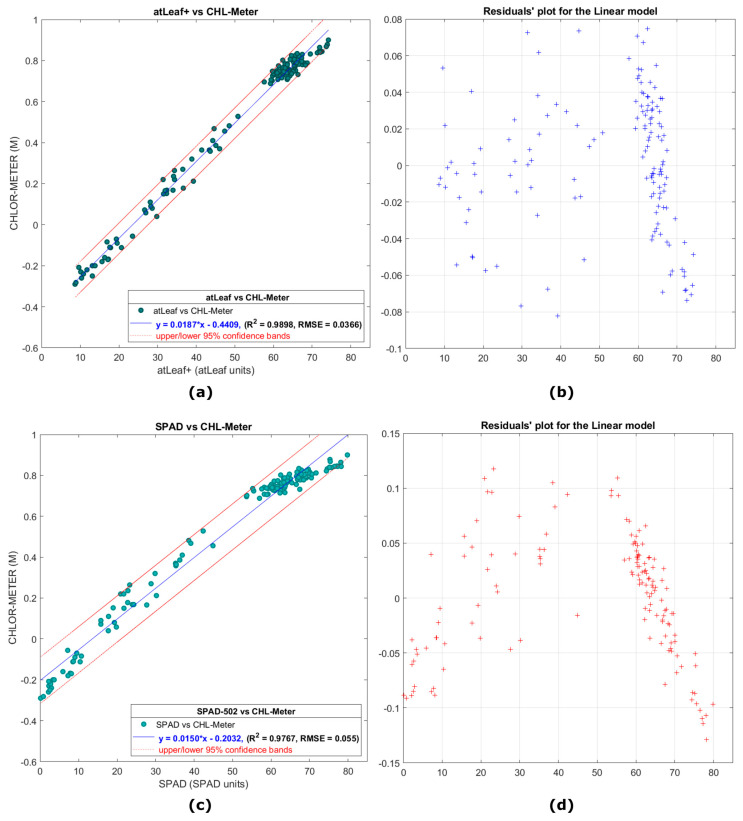
(**a**) A scatter plot showing the relation of data measurements with atLeaf CHL Plus and the proposed sensor in this work, on 30 lemon tree leaves, while in (**c**) the relation with the SPAD-502 is shown for the same data measurements. The red (dotted) lines correspond to 95% confidence prediction intervals. In (**b**,**d**), plots of the residuals (marked with +) for the simple linear regression model fit in each case applied on data plotted on (**a**,**c**) are shown, respectively.

The samples for these experiments concern measurements on 30 lemon tree leaves at different development stages (shown in Section 2.3.1, Figure 6a, and more specifically, premature, fully mature, senescent, and dry leaves, and moreover the measurements on 32 young Brussels sprouts (Figure 6b). In Figure 12a, a scatter plot is presented showing the relation of data measurements with atLeaf CHL Plus and the proposed sensor in this work. In Figure 12b, a plot of the residuals for the simple linear regression model fit applied on data and plotted on 12a is shown. It is evident that the residuals plot does not exhibit a pattern, a fact that indicates that a first-degree polynomial fit to the data, as the one applied, may be an appropriate choice. The norm of the residuals is calculated to be 0.2010. Similarly, in Figure 12c, a scatter plot is presented showing the relation of data measurements with SPAD-502 as compared to the proposed sensor in this work, on the same 30 lemon tree leaves. Respectively, in Figure 12d, a plot of the residuals for the simple linear regression model fit applied on the data and plotted on 12c is shown. It is evident, as in the previous case of lemon tree samples, that the residuals plot does not exhibit a pattern, a fact that once again indicates that a first-degree polynomial fit to the data may be appropriate. The norm of the residuals is calculated to be 0.2453.

In this work, linear regression was preferred because of its mathematical simplicity, which in turn leads to high processing speed with a minimal computational cost. As can be seen from Figure 12a,c the data measurements span a wide range of SPAD-502 and atLeaf output values, a fact that is attributed to the different development stages of leaves selected. Of the 750 leaf measurements on 30 leaves with each of the 3 sensors, the values of the atLeaf instrument ranged from 8.6 to 75.4 atLeaf units, while on the same samples the values of the SPAD-502 instrument ranged from 0 to 80.9 SPAD units. Our proposed device’s output values (by means of decimal logarithm of transmittances, $M_{CHL_{meter}}=log\left(\frac{T_{IR}}{T_{RED}}\right)$) ranged from −0.29 to 0.92. Most of the measured values fall well within the 95% interval bounds, which are also overlayed in both figures for a better visual perception of data spread. This is a positive indication and a strong possibility (95% chance) that a new similar measurement will fall within the bands. The two linear regression equations (also presented in the figures) describe a fit to the data with a strong correlation coefficient $R^{2}=0.9898$ and RMSE=0.0366 for the case of atLeaf and similarly a $R^{2}=0.9767$ and RMSE=0.055 for the SPAD-502.

**Figure 13:**
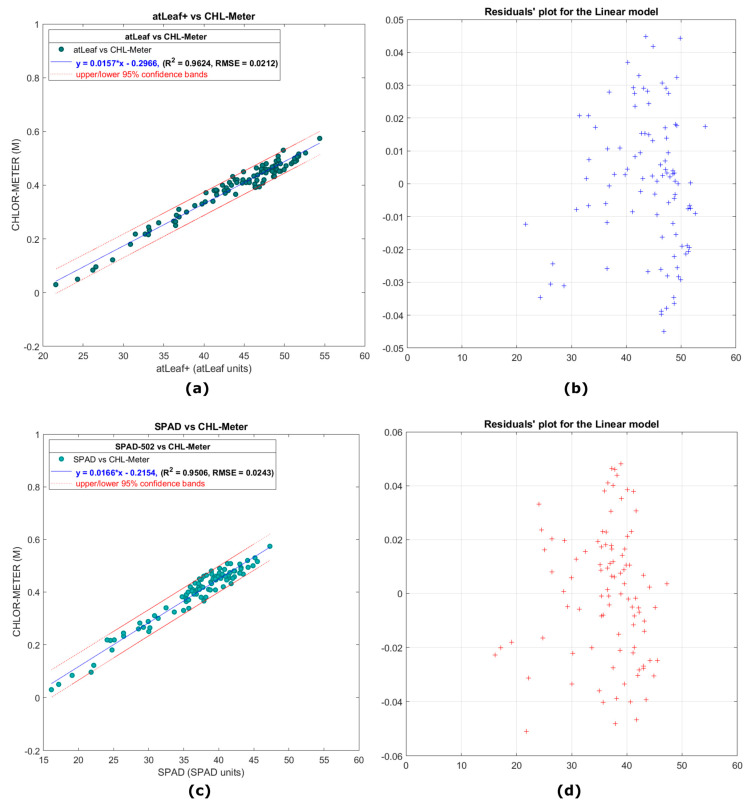
(**a**) A scatter plot showing the relation of data measurements with atLeaf CHL Plus as compared to the proposed sensor in this work, on 32 Russel’s cabbage leaves, while in (**c**) the relation with the SPAD-502 is shown for the same data measurements. In (**b**,**d**), plots of the residuals (marked with +) for the simple linear regression model fit in each case, applied on data and plotted on (**a**,**c**) are shown, respectively.

Figure 13a presents a scatter plot showing the relation of 480 data measurements with atLeaf CHL Plus and the proposed sensor in this work in the case of 32 leaves of young Brussels sprouts plants. In Figure 13b, a plot of the residuals for the simple linear regression model fit applied on data and plotted on 13a is shown. It is evident that the residuals plot does not exhibit a pattern, a fact that indicates in this experiment, too, that a first-degree polynomial fit to the data, like the one applied, may be appropriate. The norm of the residuals was calculated to be 0.2075**.** Similarly, in Figure 13c,d, the relevant scatter plot and the residuals are shown, respectively, after applying the linear model in the case of SPAD-502 and the proposed device for the same 32 leaves and 480 data measurements. The norm of the residuals was calculated to be 0.2378 for the case of SPAD-502 and the proposed device.

Of the 480 total leaf measurements on 32 leaves with each of the 3 sensors, the values of the atLeaf instrument ranged from 21.5 to 54.8 atLeaf units, while on the same samples the values of the SPAD-502 instrument ranged from 16.10 to 47.5 SPAD units. Our proposed device’s output values (by means of decimal logarithm of transmittances, $M_{CHL\_meter}$) ranged from 0.03 to 0.58. 

It is evident from the values just mentioned, but also from the resulting scatter plots and taking into account the measurement range of the atLeaf instrument, which is from 0 to 99.9 [19], and that of SPAD-502, which is −9.9 to 199 [17], that the data samples measured spanned a significantly narrower range of values than in the previous experiment. This fact was expected and is attributed to their young age and their same development stage. Nevertheless, in this experiment, the measured values fall again, as in the previous experiment, well within the 95% interval bounds. The two linear regression equations describe a fit to the data having a strong correlation coefficient $R^{2}=0.9624$ and RMSE=0.0212 for the case of atLeaf and similarly $R^{2}=0.9506$ and RMSE=0.0243 for SPAD-502. 

From the plots so far, it is evident that there is a strong linear relationship between the SPAD-502 but also the atLeaf CHL Plus, as compared each time to the proposed sensor in this work. This fact was expected, since technically speaking, all the three devices are based on similar design principles and on a similar basis of operation (similar but not exact dual-LED emission bands, transmittance measurements at similar but not exact paths through the samples, etc.).

The resulting expressions for estimating the corresponding SPAD values $\left(V_{SPAD}\right)$ as well as the atLeaf values $\left(V_{atLeaf}\right)$, given the output values of our low-cost device ($M_{RCCM}$) and considering the regression equations presented in the two Section 3.1 and Section 3.2 so far, are given in Table 4 below. These converted values can be rounded to one decimal precision point numbers, to be directly related to the ones of the commercial devices, since both follow this decimal precision (three-digit values). Moreover, the slope and the intercept coefficients of these equations were used for the conversion of units between the three devices, so that the estimation of their accuracy and repeatability could be presented in a common framework and subsequently be compared, as was shown in Section 3.1. Furthermore, relating to the plant species used in this study, for example the lemon tree leaves and the Russel’s cabbage leaves, the slope and the intercept coefficients of these developed conversion equations can be further stored in the persistence memory of the device and be recalled, if needed, during further investigation with the specific species.

### 3.3. Limitations and Comparison with Previous Studies

One of the disadvantages of the proposed device, like many other similar-in-function portable commercial chlorophyll meters (such as the SPAD-502 and the atLeaf CHL Plus utilized in this study), is the absence of a mechanism to provide a direct and universal relationship between the device’s output values and the true chlorophyll content of a leaf [35,48,49]. However, this limitation is usually treated by developing specific calibration equations among the species in order to convert the optical measurements to the real chlorophyll content [32,50,51,52,53]. The actual chlorophyll content then can be estimated indirectly, by using the greenness values acquired by the chlorophyll meters in combination with the calibration equations developed. Another limitation among these meters is the point-wise optical transmission/absorption measurements on a leaf surface, thus the calculation of the chlorophyll content is spatially limited to tiny areas. This disadvantage can be overcome by the repetitive measurements on spatially enlarged areas followed by the subsequent averaging of the results [19]. However, the great advantage of either the validated experimental (as the one proposed) or the commercial hand-held chlorophyll meter is its capability to provide an indication of leaf chlorophyll content, rapidly and with ease, under field conditions [48]. Moreover, saving the chlorophyll meter’s red, near-infrared, or any other potential wavelength transmittances that it may utilize, is a very useful feature that can be used towards a better unification of chlorophyll content estimation studies among researchers. Moreover, spectral transmittances simulations and leaf radiative models could better assist in the determination of the actual leaf chlorophyll content [35]. The proposed low-cost, experimental device presented herein delivers this simple feature in every single measurement, and also displays these intermediate results on the LCD in a fast sequence while saving them permanently. We hope that other commercial meters and non-commercial, experimental implementations follow this framework and provide this facility too.

Regarding these experimental efforts, most of them feature similar low-cost characteristics to our proposed device. Several studies relating to the design and to the accomplishment of low-cost chlorophyll meters have been realized. In [54], a device for estimating the chlorophyll content by means of leaf greenness measurements in cassava leaves is reported. The RGB TCS230 color-to-frequency converter is used in the implementation, along with an Arduino 328P microcontroller, an SD card, and a GPS module for registering data. The performance comparison is performed relative to the SPAD-502 m in 295 leaves, with the latter providing readings in the range of 4.3 to 55.8 SPAD units. While the correlation coefficient reported ($R^{2}=0.97$) is relatively strong, the referred RMSE=0.9688 is considerably higher than all the relevant results presented in the current study. Moreover, although the whole design approach seems interesting, the author admits that the developed sensor slightly underestimated RGB values at higher values. In a similar fashion in [55], the same sensor as previously mentioned, the RGB TCS230, is used for measurements of leaf greenness of lettuce plants and for nitrogen content assessment. The device is compared to the SPAD-502 m and the best correlation coefficient reported was $R^{2}=0.86$ when the leaf was placed within a 10 to 30 mm range from the sensor, while with an increased distance to 80 mm, the correlation coefficient resulted in a very low value, that is $R^{2}=0.22$ only. Moreover, the authors reported that the errors may have originated from distance dependencies as well as sunlight interferences. In [56], a hybrid, portable, slip-on design chlorophyll meter device is proposed that is paired by a Bluetooth module together with a created Android application on a mobile phone, which is used for further data visualization and data storage. The reflectance data on a leaf surface by two LED emissions are captured by a TSL250 light-to-voltage optical sensor and subsequently used for the measurements of leaf chlorophyll content using the Normalized Vegetation Index (NDVI). After 20 measurements on cassava leaves, the reported results compared to the SPAD-502 were $R^{2}=0.9681$ with RMSE=0.01413. Despite the interesting design, and the promising results reported, no details were provided by the authors regarding its circuitry. However, our belief is that the main drawback is the dependency on a mobile device to function, meaning that this device cannot function standalone. The authors in [57] present a low-cost IoT-based chlorophyll meter and compare its performance with a spectrophotometer and a SPAD-502 in maniltoa grantiflora plants. According to the authors, the device features a TSL2561 light-to-digital converter, memory, LCD and GPS modules, is of slip-on design, like the previous implementation, and can be interfaced to an IoT-based service system platform for plant fertilizer recommendations. Although the approach seems promising, and an $R^{2}=0.9705$ is reported, unfortunately no details are provided regarding the system’s functionality, regression analysis performed, the number of samples used, or the errors results. Moreover, it is not clear if this device is hand-held and portable. In [58], a low-cost and portable, greenness, six-levels color analyzer tool, targeting rice nitrogen fertilizer management, is presented. The device utilizes the optical reflectance of a single green LED, as detected by a photodiode to estimate the one out-of-six color levels (in a similar fashion to a leaf color chart [16,17]), that can be associated with rice N status estimation. The advantage of this device compared to ours, and consequently to the transmittance-based clip chlorophyll meters, such as the SPAD-502 and the atLeaf, is its reflective optical architecture that eliminates the need for cleaning both sides of the inspected leaf area prior to measurements. However, this device, although interestingly designed, is limited to only this six-color-band estimation by construction and is not meant for relative chlorophyll content measurements.

## 4. Conclusions and Future Outlook

In this study, a new, low-cost, hand-held, relative chlorophyll meter is proposed. The device was fully designed and constructed from scratch using 3D printing hardware materials and off-the-shelf simple electronics components. Although the device can operate on its own, with the methods developed it was made possible for the outcome to be well correlated to two specific and well-known commercial instruments and for them to subsequently be compared. Initial evaluation of the experimental prototype revealed promising results for measuring the relative leaf chlorophyll content. A strong linear relationship seems to correlate the output values of SPAD-502, but also those of atLeaf CHL Plus, with the proposed sensor, at least with the species tested. However, we believe that the designed and constructed methods presented in this work remain applicable to general purpose usage. The device presented is, by design and construction, capable of measuring plant leaves of maximum thickness ~1.5 mm. Although thicker samples can easily fit between the sensor clip’s upper and lower parts, these parts remain completely parallel while the samples measured remain in full darkness up to this maximum leaf thickness. In a similar fashion to the system functionality and operating principles of both the commercial instruments, the proposed device facilitates the easy navigation through the menus provided, which include, among others, the data measurements view, last data or all data measurements deletion, data averaging, as well as GPS coordinates and battery life info display. Audible sounds provided during the device’s operation facilitate the overall use of the system. Moreover, our comparative analysis demonstrated satisfactory accuracy and repeatability of our device as expressed in its own measurement system, but also when compared with the two well-known commercial instruments that we used. Although the measurements in this study were performed on two different species, we believe that our device can perform just as well in various kinds of plants and in similar measurement applications. A work in progress is the incorporation of an internal temperature sensor to be added on the clip part of the device, for ambient temperature monitoring as well as the completion of the automatic system’s calibration for “air” measurements, as an update feature. As an ongoing project, our experimental plans include the acquisition of more field datasets with the proposed device and the current instruments, but also using different modalities too, for various vegetation types and plant growth monitoring. This approach, and in combination with the parallel and systematically organized analytical chemistry and destructive laboratory procedures, will permit the methods presented here to be incorporated into a broader field of research and further validation studies of this low-cost experimental device. In this future testbed, we also aim to investigate other effects such as the effect of radiation scattering in our measurements. So far, all experiments have been performed on scattering samples, but the data from transmitted radiation ignore the impact of transmitted reflectance due to ambient scattering. In the device proposed, the sampling is performed every 10 μs and an average value of 10 sequential measurements is estimated as a basic filtering scheme of noise suppression to restrict the impact from phenomena such as transmitted reflectance. Additionally, all the measurements are related to the calculation of the relative ratios of the transmission values in red and infrared wavelengths, therefore the impact of the scattered radiation in both spectra can be assumed, without loss of generality, to be similar. Comparing measurements from the device with those from analytical experiments, we will likely be able to better understand the impact of scattered radiation in the measured data. In total, we hope that this study will facilitate a basic understanding of the commercial hand-held chlorophyll meters’ internal principles and provide a motive to growers and ordinary citizens who are interested in engaging in self-cultivation, to farmers, and to crop researchers to initiate similar studies.

## Figures and Tables

**Table 1:** Some of the transmittance-based hand-held optical chlorophyll sensors along with the wavelengths that they utilize for relative chlorophyll content measurements.

Leaf Chlorophyll Meter	LED Light Sources Wavelengths Used (nm)
Konica Minolta SPAD-502+	650, 940
FT Green atLeaf CHL PLUS	640, 940
Opti-Sciences CCM-200	653, 931
Force-A Dualex 4 Scientific	710, 850
Hansatech Instruments CL-01	660, 940
PhotosynQ MultispeQ V1.0	655, 950
Apogee Instruments MC-100	653, 931
Yara International N-tester	650, 960

**Table 2:** Correlation coefficient matrices between commercial devices and our proposed one.

Chlorophyll Meters	Lemon Tree (*r*)	Young Brussels Sprouts (*r*)	Blue and Green Filters (*r*)	Green Filters (*r*)
AtLeaf—proposed device	$r_{1}=\left[\begin{array}{cc} 1 & 0.9949\\ 0.9949 & 1 \end{array}\right]$	$r_{1}=\left[\begin{array}{cc} 1 & 0.9810\\ 0.9810 & 1 \end{array}\right]$	$r_{1}=\left[\begin{array}{cc} 1 & 0.9805\\ 0.9805 & 1 \end{array}\right]$	$r_{1}=\left[\begin{array}{cc} 1 & 0.9970\\ 0.9970 & 1 \end{array}\right]$
SPAD-502—proposed device	$r_{2}=\left[\begin{array}{cc} 1 & 0.9883\\ 0.9883 & 1 \end{array}\right]$	$r_{2}=\left[\begin{array}{cc} 1 & 0.9750\\ 0.9750 & 1 \end{array}\right]$	$r_{2}=\left[\begin{array}{cc} 1 & 0.9865\\ 0.9865 & 1 \end{array}\right]$	$r_{2}=\left[\begin{array}{cc} 1 & 0.9872\\ 0.9872 & 1 \end{array}\right]$

**Table 3:** Accuracy and repeatability as estimated in this study and as compared to the manufacturers’ manual, where applicable.

	*Manufacturer’s Manual* [23,25]		*Estimated Values in This Study*	
Chlorophyll Meter	Accuracy	Repeatability	Accuracy	Repeatability
atLeaf CHL Plus (atLeaf units)	[−0.6, +0.5]	0.054 *	[−0.7, +0.6]	0.0218
SPAD-502 (SPAD units)	±1.0 **	±0.3 **	[−0.6, +0.9]	0.06
			[−0.03, +0.02]	0.0044
Proposed device (CHL-meter units)	n/a	n/a	±1.34 (atLeaf-units)	0.2999 (atLeaf units)
			±1.22 (SPAD units)	0.2780 (SPAD units)

* Repeatability on a green 50.2 ±0.1 [25]. ** SPAD value between 0.0 and 50.0 [23]

**Table 4:** Conversion equations to SPAD and atLeaf values.

Samples Measured	atLeaf CHL Plus Conversion Values *	SPAD-502 Conversion Values *
Lemon tree leaves	$V_{atLeaf}=53.476\times M_{CHL\_meter}+23.577$	$V_{SPAD}=66.667\times M_{CHL\_meter}+13.547$
Brussels sprouts leaves	$V_{atLeaf}=63.694\times M_{CHL\_meter}+18.892$	$V_{SPAD}=60.241\times M_{CHL\_meter}+12.976$
Non-leaves	$V_{atLeaf}=67.567\times M_{CHL\_meter}+19.16$	$V_{SPAD}=62.893\times M_{CHL\_meter}+13.27$

* Conversion to estimated values, given the output values of the proposed device ($M_{CHL\_meter}$).

## Data Availability

The data used to support this study’s findings are available from the corresponding author upon logical request.
